# UK Clinical Guideline for Best Practice in the Use of Vaginal Pessaries for Pelvic Organ Prolapse

**DOI:** 10.1002/nau.70306

**Published:** 2026-06-22

**Authors:** Claire Brown, Angie Rantell, Kate Anders, Lucy Brett, Avril McDowell, Di Tilston, Nicola Wilson, Julia Wilkens, Naz Abbas, Lucy Dwyer, Georgia Smith, Kate Lough

**Affiliations:** ^1^ THIS Institute and University of Cambridge Cambridge UK; ^2^ King's College Hospital London UK; ^3^ Ashford & St Peters Hospital Foundation Trust Surrey UK; ^4^ Lived Experience Participant; ^5^ Wigan and Leigh NHS Trust Wigan UK; ^6^ NHS Lothian Edinburgh UK; ^7^ Manchester University NHS Foundation Trust Manchester UK; ^8^ The Warrell Unit, Saint Mary's Hospital Manchester University NHS Foundation Trust Manchester UK; ^9^ Gloucestershire NHS Trust Gloucester UK; ^10^ Chair of Pelvic Obstetric and Gynaecological Physiotherapy UK


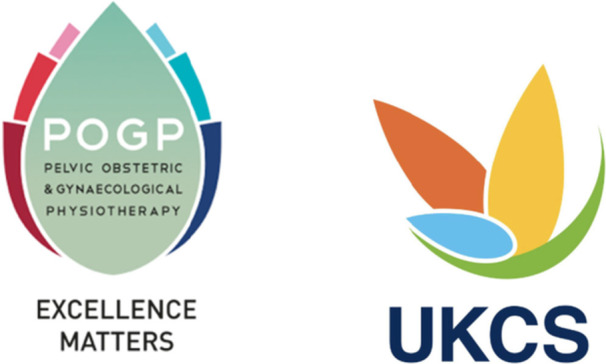


This clinical guideline was developed in response to the publication of the shared priorities of women and healthcare professionals for future research in pessary use for prolapse, in 2018 [1]. The many uncertainties about pessary use were highlighted and the absence of a standardized, evidence‐based UK guideline was identified (Appendix A).

Members of the UK Clinical Guideline Group represented key stakeholders and pessary users.

This guideline was originally published with stakeholder approval or support from the following organizations:

- British Association of Urological Nurses (BAUN)
- International Urogynecological Association (IUGA)
- Pelvic Obstetric and Gynaecological Physiotherapy (POGP)
- Scottish Pelvic Floor Network (SPFN)
- The Association of Continence Professionals (ACP previously ACA)
- The British Society of Urogynaecology (BSUG)
- The Chartered Society of Physiotherapy (CSP)
- The Pelvic Floor Society (TPFS)
- The Royal College of Nursing (RCN)
- The Royal College of Obstetricians and Gynaecologists (RCOG)
- The United Kingdom Continence Society (UKCS)
- The Royal College of General Practitioners (RCGP) 2025

Ongoing stakeholder approval is being sought.

*Review*

This guideline will be due for review in 2028.

## 1 Aims

This consensus document aims to deliver a best practice guideline for healthcare professionals providing nonsurgical management for pelvic organ prolapse using vaginal pessaries, and information for women seeking or being offered this treatment option. The guideline is suitable for all healthcare professionals, and it may be used in different ways due to differences in levels of experience and training. Recommendations are based on evidence where available, or expert opinion derived from clinical consensus within the guideline group, as indicated in the guideline.

The multidisciplinary UK Guideline aims to:

- standardize pessary management
- develop and widely publicize information for pessary users to inform, empower and manage expectations;
- identify key barriers to optimal pessary management and offer possible solutions
- develop a training competency framework for healthcare professionals with recommended levels for supervision, observation and assessment of competence (Appendix B):
- provide recommendations for data recording and audit, and as a consequence, facilitate future research; and
- maintain an established group to review future and emerging evidence.

The guideline is not intended as a substitute for advice and training from an appropriate healthcare professional and is expected to be part of a patient—centered treatment program for pelvic organ prolapse.

## 2 Equality, Diversity, and Inclusion

The UK Clinical Guideline Group are not aware of any adverse impact on protected characteristics with the implementation of this guideline. The guideline does not affect the equality, diversity and inclusion policies in place in the individual organizations that implement the guidance within a pessary provision service.

## 3 Terminology

The term “pelvic organ prolapse” will be used throughout this document, and shortened to “prolapse” for clarity if required. The term “vaginal prolapse” was considered more helpful by the pessary users and is used in the patient information sections.

The term “vaginal pessary” will be used throughout the document and shortened to “pessary” for clarity if required.

The term “woman” is used throughout this document to represent any person with symptoms of pelvic organ prolapse who may be seeking management that includes trial of a pessary.

The term “pessary user” will be used for those who are seeking to trial or manage their prolapse symptoms with a pessary either on a clinic ‐based or self‐management basis.

## 4 Best practice guideline

### 4.1 Introduction

*Pelvic organ prolapse* is defined as an “anatomical prolapse with descent of at least one of the vaginal walls to or beyond the vaginal hymen with maximal Valsalva effort WITH the presence either of bothersome characteristic symptoms, most commonly the sensation of vaginal bulge, or of functional or medical compromise due to prolapse without symptom bother.” [2]. A vaginal prolapse may affect the front (anterior), back (posterior), or top (apical) sections of the vagina.

Common symptoms are:

- vaginal heaviness and bulge;
- bladder and bowel difficulties that may include urgency, frequency, leakage and incomplete emptying; and
- discomfort that may be felt vaginally, abdominally, or during sexual activity and may include low back pain.

Many women may experience bladder, bowel and sexual symptoms that could be associated with but not caused by the prolapse. Some 20%–40% of all women will experience prolapse symptoms that may be bothersome and affect their quality of life [3, 4, 5].

Pelvic organ prolapse is measured on clinical examination and staged or graded according to the extent of downwards displacement (descent) of the most‐affected vaginal compartment. The level of descent does not necessarily correlate to the symptoms experienced.

## 5 Treatment

Management of a bothersome pelvic organ prolapse should be a shared decision‐making process to help a woman achieve symptom reduction. Pelvic organ prolapse treatment options include:

- watch and wait—the prolapse may not worsen over time
- non‐surgical management that may include supervised pelvic floor muscle training, use of a vaginal pessary and lifestyle modifications to reduce symptoms experienced for example, weight loss and exercise advice; bladder and bowel symptom management and optimsing vaginal health that may include the use of topical oestrogen as clinically indicated
- surgery—aiming to restore the vaginal anatomy

NICE Guideline NG123 [6] recommends that a vaginal pessary for women with symptomatic pelvic organ prolapse should be considered, alone or in conjunction with supervised pelvic floor muscle training. A woman who has chosen to try pessary treatment should be referred to a urogynaecology service if pessary care services are not available locally.

*Vaginal pessaries* are used intravaginally to try to restore the prolapsed organs to their normal position and relieve symptoms. They are usually made of plastic or silicone and are available in a range of types and sizes and may have additional features to help with stress urinary incontinence, and more supportive options. A ring pessary is the most commonly used and usually the first to be tried. Choice and fit of pessary are based on clinician experience, availability, whether the woman wants and can self‐manage and/or to be sexually active with penetration, and which type of pessary is retained and comfortable. The fitting process is trial and error and several different sizes and types may need to be tried over several appointments before the woman is comfortable during all activities of daily life, able to pass urine with the pessary in place or confident with self‐management.

## 6 Pessary Use for Symptomatic Pelvic Organ Prolapse Best Practice Guidance

The clinical pathway for pessary provision and management (p. 13) follows the stages outlined below.

### 6.1 Indications

A pessary may be offered to a woman of any age:

- for short‐ or long‐term management of bothersome symptoms of pelvic organ prolapse if she has a preference for or is willing to agree to a trial of pessary use, there are no contraindications (see below), and she understands that regular attendance for follow‐up is required unless self‐management is chosen and appropriate support is available (evidence)
- who has not completed her family and needs an interim solution for symptomatic prolapse until surgery, if indicated, can be considered at a later date (evidence); or
- as part of the assessment process for associated bladder and bowel symptoms that may be relevant where surgery is planned. This may be a short term option or the woman may opt to continue with a pessary and defer surgery (evidence).

### 6.2 Contraindications

A pessary should not be considered in the following situations:

- The woman is neither able to comply with regular follow‐up, nor self‐manage the pessary (evidence).
- There is active vaginal or pelvic infection, inflammation, unexplained bleeding, or ongoing vaginal or cervical cancer (evidence).
- The vaginal tissue is severely atrophic and has not responded to pre‐pessary oestrogen treatment (evidence).
- The vaginal dimensions make fitting too difficult (expert opinion).
- There is identifiable synthetic vaginal mesh erosion (expert opinion).

### 6.3 Caution Required

A pessary may be an option, but additional caution is required in the following situations:

- poor vaginal health requiring vaginal oestrogen therapy prior to a pessary fitting (evidence);
- the woman has had previous radiotherapy affecting the vaginal tissues (evidence)
- a synthetic mesh has been placed in the vagina during previous surgery (expert opinion); and/or
- pre‐existing vaginal pain (expert opinion) (e.g., pudendal neuralgia)
- the woman is immunosuppressed (expert opinion)

### 6.4 Complications

Vaginal changes are common following pessary use but do not always indicate that pessary use should stop. Known complications include:

- new bladder and bowel symptoms; these may include occult stress urinary incontinence due to reduction of prolapse, or urgency for voiding or defecation, urinary retention/obstructed defecation due to the pressure effects from the pessary) (evidence].
- vaginal ulceration is uncommon but may require biopsy if it fails to heal (evidence).
- difficulty with removal is uncommon for a ring pessary and more common for others (expert opinion);
- incarceration is an uncommon risk where the pessary is displaced from its original position and becomes embedded in the vaginal or cervical tissues) (evidence)

The following table is based on the GMC Professional Standards Decision‐making and Consent document [7] and presents the known complications related to use of vaginal pessaries (Table 1).

**Table 1:** Complications related to the use of vaginal pessaries.

Complication	Term	Equivalent numerical ratio	Colloquial equivalent
Increased vaginal discharge	*Very common*	1/1 to 1/10	A person in a family
Erosion or abrasion of vaginal skin	*Common*	1/10 to 1/100	A person in a street
Vaginal bleeding			
Discomfort			
Pessary expulsion			
New bladder or bowel symptoms			
Vaginal ulceration	*Uncommon*	1/100 to 1/1000	A person in a village
Difficulty with removal			
Infection			
Incarecration			
Fistula (serious complications)	*Rare*	1/1000 to 1/10,000	A person in a small town
Cancer	*Very rare*	Less than 1/10,000	A person in a large town

#### 6.4.1 The Clinical Pathway for Pessary Use for Prolapse


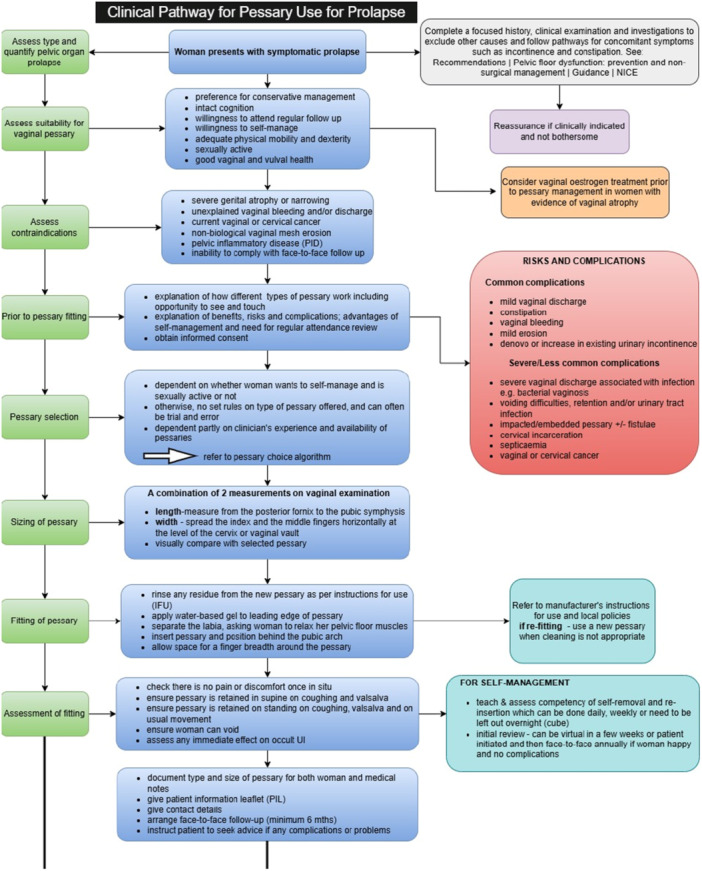



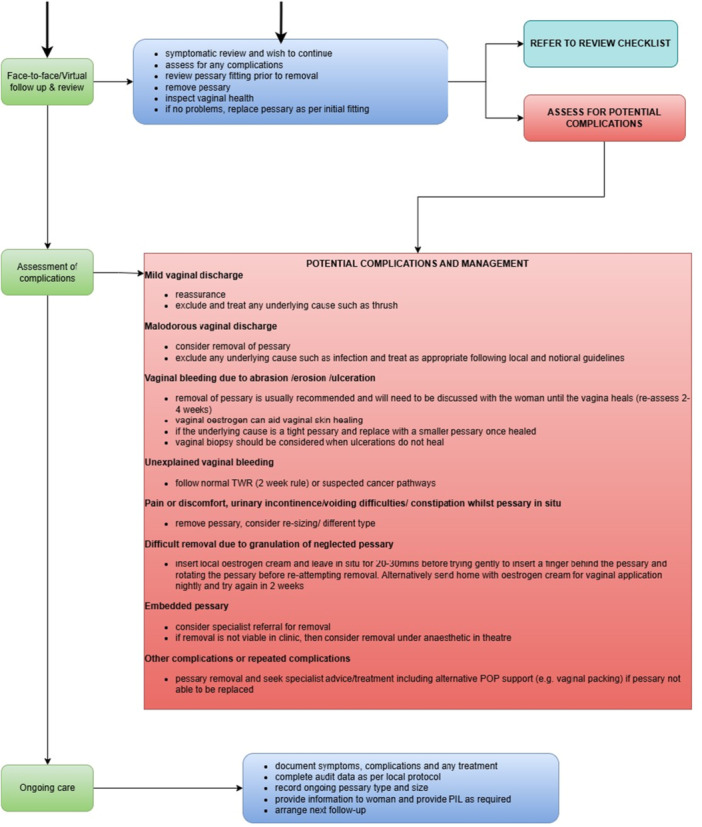


### 6.5 Pessary Fitting

If a woman chooses a trial of pessary once treatment options for prolapse have been described and discussed, and informed consent obtained, she will need assessment to confirm her suitability for this option. If she still wishes to proceed, a suitable pessary will be fitted according to the clinical practice pathway above (p. 13) and the pessary choice flowchart below (p. 16).

### 6.6 Pessary Choice and Indication

Vaginal pessaries can be broadly divided into three categories:

- support pessaries (ring, ring with support, Gehrung, Hodge, Dish, and Shaatz)
- pessaries with stems (shelf and Gellhorn)
- space‐filling pessaries (donut, cube and inflatable).

This document will refer to pessaries by name not category.

Pessaries vary in size, shape, and material. Detailed information about the shape, fitting process, and any indications for use specifically related to the design are included in Appendix C for all pessaries employed in routine clinical practice.

Information about the fitting and removal of the most commonly used pessaries is provided within the Pessary Choice Flowchart below.

#### 6.6.1 Pessary Choice Flowchart


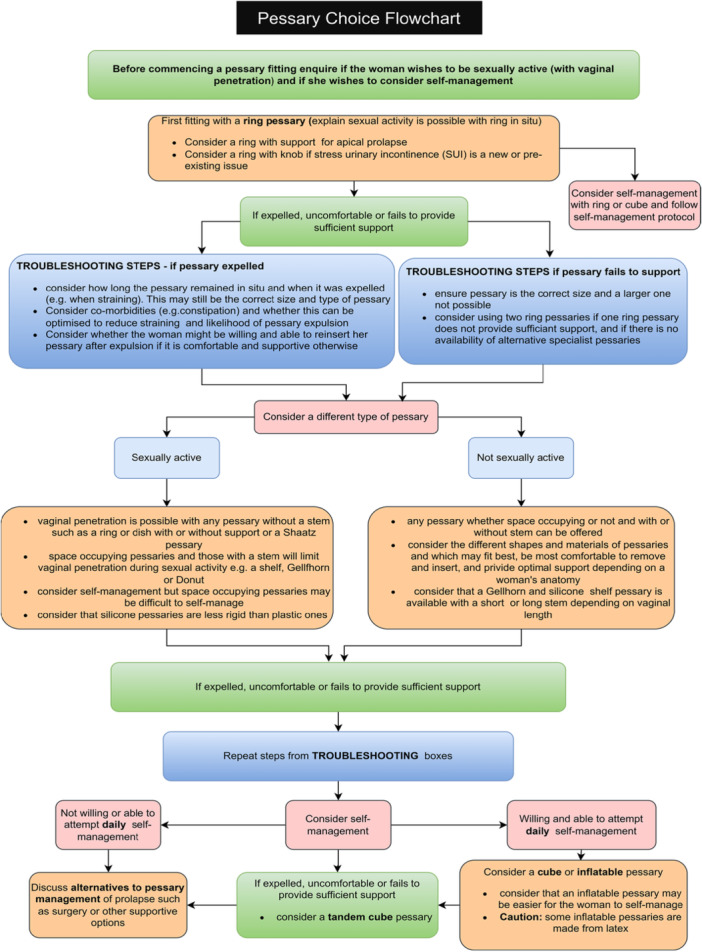


### 6.7 Pessary Review—Time Scales and Changes

The evidence for frequency of follow‐up is not conclusive. It is generally agreed that after the initial fitting of a pessary that a review appointment at 4–6 weeks should be offered. This can be carried out either in‐person or remotely depending on local practice although face to face follow‐up is preferred and allows for regular vaginal examination to check for complications. In addition to this pessary users should have details of who to contact should they have issues with their pessary prior to their routine appointments. Good practice is that the follow‐up interval should be no longer than 6 months for pessary users with clinic‐based care, while a pessary is still in use (expert opinion).

Pessary users who choose to self‐manage their pessary should be advised to remove the pessary at least every 6 months; daily removal is advised for cube pessary users. Self‐managing women should receive a follow‐up appointment at a minimum of every 18 months. Pessary users who are self‐managing their pessaries should also have access to patient initiated follow‐up should any issues arise prior to their 18 month follow‐up. Vaginal examination is important in ongoing pessary care (evidence).

Recommendations regarding types of pessaries and their suitability for self‐management can be found in Appendix C.

A new pessary is required when the pessary is showing signs of wear, such as cracks or splits, is not holding its shape to support the prolapse sufficiently, or in accordance with any specific manufacturer's instructions about replacement guidance [expert opinion].

At each visit satisfaction with pessary management of prolapse symptoms and the fit of the pessary should be assessed. Other options for management of prolapse should also be discussed to ensure that continued management with a pessary remains appropriate The pessary should be removed, and the vaginal walls, and if present, the cervix, inspected using a speculum. The same pessary may be washed and replaced in accordance with manufacturer guidance if it does not show signs of wear, or, a new one fitted if required (p. 19) (expert opinion).

All clinical findings, and the size and type of pessary inserted, should be clearly documented and copy of this information should be given to the pessary user. See the Pessary Review Checklist below:

### 6.8 Pessary Review Checklist


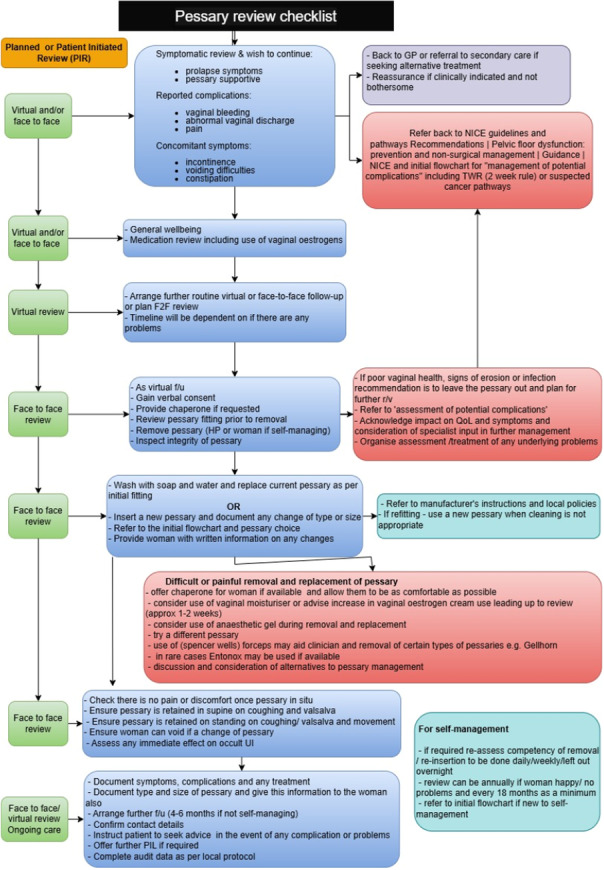


### 6.9 Use of Vaginal (Topical) Oestrogen

Vaginal atrophy manifests as thinner, drier, more fragile and less‐elastic vaginal tissue, and may occur when there is a reduction of circulating oestrogen, such as after the menopause or during breast‐feeding. As an intravaginal device, a pessary sits against the vaginal walls and may cause abrasions. A vaginal pessary can excoriate the atrophic skin, causing discomfort. To minimize the effect of this, a woman with vaginal atrophy considering pessary use, including a woman who is taking systemic hormone replacement therapy (HRT), can be prescribed vaginal oestrogen prior to, or during, pessary use. Vaginal oestrogen is administered as a cream, a vaginal tablet or pessary, or as a vaginal ring containing a controlled release delivery system lasting 3 months (evidence).

Vaginal atrophy is likely to respond to oestrogen treatment within 3 months, but an individual's response, local practice and specific products may vary. A follow‐up vaginal examination will determine whether the vaginal skin has improved sufficiently to allow pessary use with or without ongoing oestrogen support. The management of vaginal atrophy may require the lifelong use of vaginal oestrogen (expert opinion).

For a woman where systemic HRT is contraindicated, vaginal oestrogen may be considered, after seeking advice from a healthcare professional with the relevant clinical expertise. A woman with vaginal dryness may also use moisturizers and lubricants alone or in addition to vaginal oestrogen (evidence). Pessary practitioners should be aware of the recognized contraindications and cautions relating to the use of vaginal oestrogen which are clearly stated in the NICE CKS Intravaginal oestrogen [8].

For those who do not improve with moisturizers or lubricants, and vaginal oestrogen is contraindicated, alternative treatment options of vaginal atrophy may include vaginal DHEA (dehydroepiandrosterone), selective oestrogen receptor modulators and laser therapy. Further information may be found in the BMS Urogenital atrophy recommendations [expert opinion] [9].

## 7 Additional Considerations for Pessary Use in Prolapse

### 7.1 Pessary Self‐Management

It is recommended that any woman who has been assessed, found suitable, and has agreed to try a vaginal pessary, should be offered the possibility of self‐management if this option is supported and available. This puts the woman in control of managing her own condition, to suit her lifestyle [evidence].

To self‐manage successfully, the woman needs to become confident about inserting, removing and cleaning the pessary, and be able to order a new one, book and attend follow‐up appointments, and ask for advice as needed. All the elements of successful self‐management can be taught by the pessary practitioner with verbal information given together with written guidance, including contact details in case an earlier appointment is required [evidence].

Pessary self‐management is common with ring and cube pessaries, but may be more challenging with other types of pessary for example the Gellhorn, shelf and Donut pessary. [expert opinion]. Please refer to the Pessary Choice Flowchart (p. 16). Women with any degree of cognitive impairment (e.g., dementia) need to be individually assessed for a self‐management pathway (expert opinion).

For training in self‐management please refer to Appendix B, standard 7.

This guideline document includes a Patient Information section with Infographics, FAQs, Self‐management PIL and Leaflet (Appendix D).

### 7.2 Patient Information

The minimum information that is required for a woman choosing a trial of pessary, or for ongoing pessary management in clinic or self‐management is (expert opinion):

- that the fitting process may require several different pessaries or sizes to be tried before a successful fit is achieved
- that the woman has been shown and understands where the pessary will be positioned and what it should feel like
- that the bladder and bowel functions should not be negatively affected although there may be an increase in stress urinary incontinence
- mild postfitting discomfort may be experienced but will pass in a day or two
- that the woman is clear about the follow‐up process including managing unexpected complications or routine care arrangements
- that the woman has had time to ask questions, is comfortable and has passed urine before leaving the clinic
- that the woman has information to take away including details of the pessary in place, a review appointment if planned and a contact number to call if any problems
- an accessible patient information leaflet should be provided as best practice

## 8 Vaginal Devices Procured Independently

This guideline does not provide information about vaginal devices procured outside of a healthcare setting. While some pessaries available for private purchase by individuals are identical to those used in a healthcare setting the guideline committee are aware of instances where products have been marketed as “pessaries” without undertaking the necessary quality or safety checks required of a medical device. For this reason the guideline group would caution the purchase of any devices outside of a healthcare setting because the level of safety and clarity of instructions for use and the required medical oversight is not guaranteed.

## 9 Pessary Service Specification

The pessary service specification will depend on location of the service for example, primary, secondary or tertiary care and, to some extent, by the numbers of patients to be seen. Administrative requirements for service provision and set up will also vary depending on location and clinical systems in use in that area. However, the following points need to be incorporated (expert opinion):

Access, capacity, and service availability

- *Clinical environment*:
  ◦ Pathway/access to microbiology, imaging services, and MDT referral

- *Administration/clinic structure*:
  ◦ Clear referral pathways to pessary provision
  ◦ Supporting MDT pathway
  ◦ Ensuring that the number of sessions per week/month meet the service demands/workload
  ◦ Creating capacity for face‐to‐face appointments to ensure regular follow‐up

Safety, risk, and safeguarding

- *Clinical environment*:
  ◦ Emergency planning/access to resuscitation equipment
  ◦ Risk assessments should be carried out for infection control, lone worker and manual handling

- *Administration/clinic structure*:
  ◦ The local policy for safeguarding, intimate examinations, chaperoning and manual handling should be followed
  ◦ Availability of chaperones

Privacy, dignity, and examination support

- *Clinical environment*:
  ◦ Maintenance of privacy and dignity
  ◦ Patient changing area
  ◦ Appropriate examination couch or chair
  ◦ Additional lighting for speculum examinations

Equipment, materials, and consumables

- *Clinical environment*:
  ◦ Space for equipment/consumable storage, and consultation, as well as accessibility for wheelchairs/hoists
  ◦ Access to medication and safe storage for example, local anaesthetic, topical oestrogen
  ◦ Access to toilet facilities and waiting area with water for patients
  ◦ Appropriate disposal facility for clinical waste
  ◦ Access to decontamination services if fitting kits being used

- *Administration/clinic structure*:
  ◦ Management of materials/consumables
  ◦ Patient information leaflets, ensuring the supply of reliable and consistent, up to date material
  ◦ Provision of information on pessary insertion and ongoing management and how to contact the service if concerns noted

Follow‐up and continuity of care

- *Administration/clinic structure*:
  ◦ Availability of short notice follow‐up appointments to assess and manage pessary associated complications
  ◦ Adequate staffing to include contingency planning/continuity of service
  ◦ Booking of pessary appointments of appropriate length—recognizing that pessary fittings and teaching self‐management will take longer than most routine pessary reviews, but also noting variations in complexity due to patient and pessary factors that may require extended appointments
  ◦ Administration, including processing of clinical notes, letters and notifications to be sent to the primary care provider
  ◦ Establishment and maintenance of a pessary database noting all patients in the service and the size and type of pessaries used
  ◦ Clinic coding
  ◦ Facilitation of audit and service evaluation

## 10 Regulatory Guidance for the Use of Pessaries

Pessaries are classified as medical devices for vaginal use. The classification of the device and the approved Instructions For Use (IFU), determines the licensed use of the product. Pessary practitioners are required to understand the IFU for the device that they are fitting and provide the correct information about device management. If pessaries are not supplied in accordance with the specified IFU, they are being used “off‐label” and this information should be included in the patient notes and patient information. The pessary practitioner is responsible for the use of a product outside the licensed remit.

### 10.1 Pessary Clinic Staffing

Pessary services in the United Kingdom are delivered in a variety of models by a variety of healthcare professionals, but all have the same common principles (expert opinion):

- Patient safety and well‐being necessitates there being an appropriately trained HCPs and a suitably trained assistant/chaperone available for every patient
- All staff need to be aware of local policies including infection control, manual handling, intimate examination, and chaperoning
- All staff demonstrate competence as set out in the guidelines for example, clinical assessment and pelvic examination, pessary fitting, pessary evaluation and management of complications, teaching self‐management
- Opportunities for self‐management or clinic based care dependent on patient preference or pessary need
- Pathway for protocol available to guide practice
- It is recommended that HCPs are encouraged to identify their level of competency and work to extend their scope of practice

### 10.2 Monitoring and Audit

It is expected that the impact of this guideline will lead to a more consistent pessary provision across the United Kingdom and in turn an opportunity to improve the collection and audit of pessary related data. The guideline group recommends that data is collected and collated for:

- patient satisfaction using a reliable, replicable, and simple Patient Reported Outcome or Experience Measure (PROM or PREM), such as the Patient Global Impression of Improvement (PGI‐I)
- pessary effectiveness using a validated PROM or PREM available studies suggest the following are effective for prolapse ICIQ‐VS, PFDI, PFDI‐20, PFIQ and POP‐SS)
- review times for follow‐up and replacement
- self‐management: offered; declined; accepted; success
- pessary data: type and cost
- adverse events and significant complications

## Ethics Statement

The authors have nothing to report.

## Consent

The authors have nothing to report.

## Conflicts of Interest

L.D.: Completed doctoral research and is a co‐applicant in research related to pessary use for prolapse funded by the NIHR/HTA; received payment to teach on the Mediplus Advanced Pessary Care study day.

A.R.: Honorarium received for teaching for Mediplus.

J.W.: Taught at a Mediplus study day (unpaid) and has been an organizer for study days and conferences for Mediplus.

## Figures and Tables

**Figure B1:**
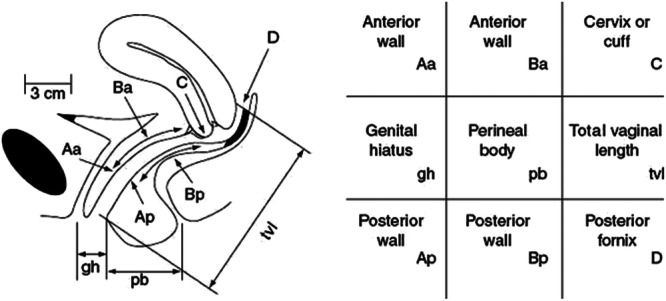
Measurement points for POP‐Q system.

**Figure C1:**
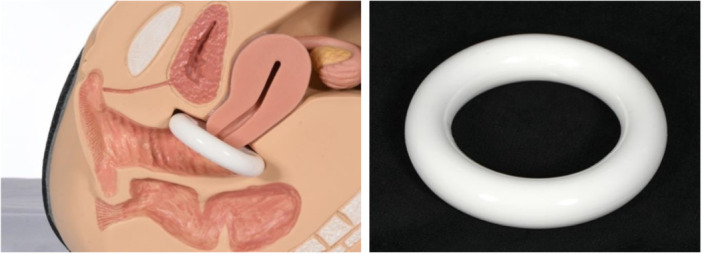
Ring pessary in place.

**Figure C2:**
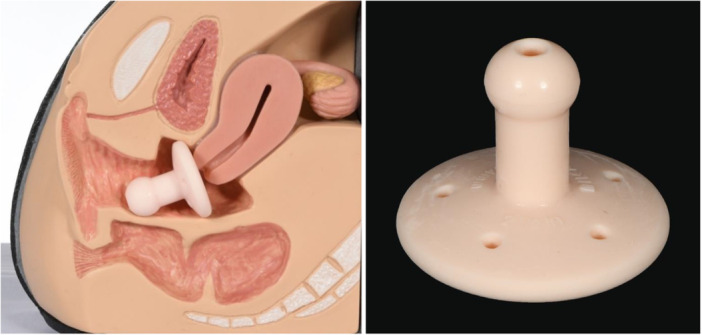
Gellhorn pessary in situ.

**Figure C3:**
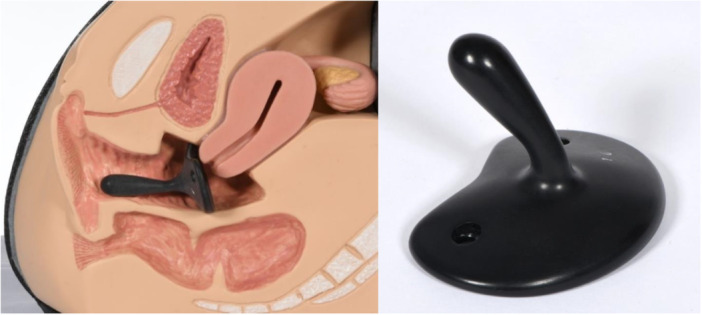
Shelf pessary in situ.

**Figure C4:**
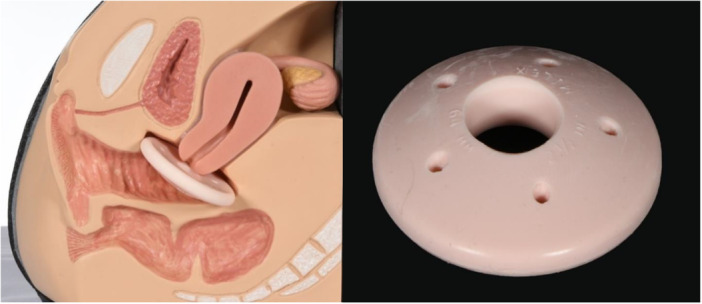
Shaatz pessary in situ.

**Figure C5:**
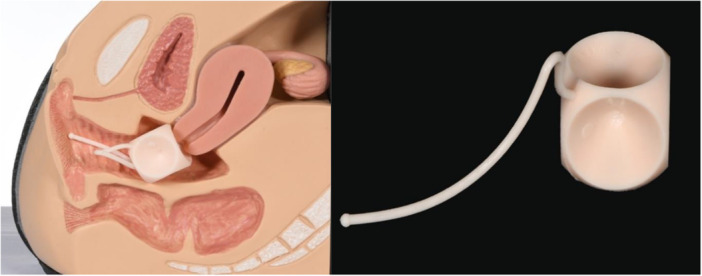
Cube pessary in situ.

## Data Availability

Data sharing not applicable to this article as no data sets were generated or analyzed during the current study.

## Appendix A

**Development of the UK Clinical Guideline Group for the use of pessaries in pelvic organ prolapse**

The 2020 UK Clinical Guideline Group included healthcare professionals and pessary users with experience of clinic‐based care and self‐management. The health professionals provided representation from the specialities of gynaecology, urology, urogynaecology, obstetrics, colorectal, specialist physiotherapy in pelvic health and specialist nurses in urogynaecology. The 2025 review group did not include urology and colorectal representation.

The members of the group were:

Angie Rantell, Consultant Nurse, Urogynaecology, King's College Hospital

Kate Anders, Lead Nurse for Urogynaecology, Ashford & St Peters Hospital Foundation Trust

Lucy Brett, Lived experience participant

Avril McDowell, Lived experience participant

Di Tilston, Lived experience participant

Dr Kate Lough, Chair POGP, specialist physiotherapist in pelvic health

Claire Brown, PhD student THIS institute and University of Cambridge, specialist physiotherapist in pelvic health

Nicola Wilson, Urogynaecology Consultant, Wigan and Leigh NHS Trust

Dr Julia Wilkens MD Consultant Subspecialist and Lead for Urogynaecology in NHS Lothian

Naz Abbas, Clinical Research Fellow, Manchester University NHS Foundation Trust

Lucy Dwyer, Consultant nurse, The Warrell Unit, Saint Mary's Hospital, Manchester University NHS Foundation Trust

Dr Georgia Wilson, Consultant Obstetrician & Gynaecologist, Gloucestershire NHS Trust

As part of the development work towards this Guideline, the James Lind Alliance pessary use for prolapse priority setting partnership identified a top 10 future research needs. The top three were:

- How might a pessary affect sexual activity?
- Do pessaries have an effect on the psychological well‐being of women?
- What is important for a pessary self‐management program?

The 2025 review has been informed by new research that has been published since 2018 following a rapid evidence review conducted in January 2025 (Appendix E). The pessary guideline group undertook a collective review of the newly identified evidence and evaluated its relevance to the guideline document. This process involved multiple rounds of discussion and decision‑making, after which it was concluded that the new research does not provide evidence for any significant change of practice contained within this guideline but does strengthen the clinical rationale for promoting self‐management of pessaries where possible and supported by the healthcare provision arrangements. The TOPSY study published in 2023 concluded that pessary self‐management is cost‐effective, does not improve or worsen quality of life compared to clinic based care, and has a lower complication rate. Additionally the randomized controlled trial found that women in the self‐management group however did have greater self‐efficacy in relation to managing problems associated with their pessary, and, thus more confidence in their ability to remove and replace their pessary.

The published studies indicates that future research is required in the following areas:

- What is the best way to measure the effectiveness of a pessary used to treat prolapse?
- Does a pessary used for prolapse have an effect on the severity of the prolapse over time?
- What is the optimal management pathway for a woman using a pessary to maintain effectiveness, reduce the likelihood of dissatisfaction and prevent complications?
- Does the type of pessary make a difference to the outcome of the treatment?
- What are the experiences of women who use a pessary?
- What are the mechanisms by which self‐management reduces complication rates, and does it leads to a reduction in pessary discontinuation.
- Uptake and offer of pessary management in ethnic minority populations
- A validated pessary complications questionnaire needs to be developed to enhance the rigor and consistency of future trials of pessaries.
- Future research is needed to focus on models of pessary self‐management follow‐up, for example, should a pessary review be woman‐initiated or does it require to be planned at specific intervals?
- What determines pessary selection in clinical practice
- What is the optimal training program for clinicians acquiring pessary management competencies.
- What constitutes appropriate patient education related to pessary use and management.

The 2025 Clinical Guideline was shared with the stakeholders listed on page 2 for review and ongoing endorsement.

The suggested changes were implemented.

*Feedback*: Users of this document can provide feedback using this link UK Pessary Guidance Document Feedback Form—Fill in form

## Appendix B

**Training framework and standards**

Clinicians offering pessary management must comply with their own professional standards to ensure that they are working within their own scope of practice and they receive training that is compliant with professional guidance.

Before undertaking any training in the use and management of vaginal pessaries, the clinician must have completed training in vaginal examination and be using this in clinical practice. A baseline knowledge and experience should already be established.

The below algorithm allows clinicians to identify where their current skill set is currently and identify what standards are required to add pessary skills to their scope of practice. Entry points are included for:

- New clinician with no experience of prolapse assessment
- New clinician with experience of prolapse assessment
- Existing clinician with ring pessary change only
- Existing clinician with ring fitting and change only
- Existing clinician with ring and other pessary change only
- Existing clinician with ring and cube fitting only

*Entry level training algorithm*

*Aims of the training document*

This document aims to serve as a framework for clinicians who wish to achieve pessary skills by following the standards. We acknowledge variation in pessary services and differing requirements to fulfill job roles. The document has been designed to be flexible to facilitate the learning process aimed to suit variations in services and job roles, but also aims to standardize pessary clinicians' skill level.

Consequently, this document may act as a personal logbook for the clinician to accumulate signatures to demonstrate their achieved competence in pessary care and prove fitness to practice.

It has been recognized by The Centre for Advancing Practice for Pelvic Health Advanced Practice Area Specific Capability and Curriculum Framework [10] a portfolio of work is required to record learning activities and feedback from others, capture critical reflection on learning progression, and a clinician should articulate critical engagement with, and use of, the evidence base in their learning and practice. This training document should be used as a platform to document these requirements.

All woman treated by a clinician during training and being observed by their supervisor, should be informed of their training status.

*Consent*

Consent should be obtained after the woman is fully informed, involved in the discussion and reassured about the process of a vaginal examination. Enough time should be given for the woman to ask questions.

Information given should include:

- the reason for the examination, including the use of a speculum and lubrication
- the duration of the examination
- discuss the positions that may be required during the examination
- discuss what may be expected of the woman, for example straining/bearing down and how to achieve this
- explain straining/bearing down during the examination may cause leaking or wind
- reassure that any pain/discomfort or bleeding experienced should be minimal and very temporary

After the examination, findings should be explained and all treatment options discussed.

*Competency assessment*

For each learning objective within the standards described, there is the opportunity for the pessary clinician to evidence their learning and clinical competence. An assessor may sign which level they deem the clinician to be practising at for each particular objective. An assessor should be someone who has relevant pessary skills themselves and feels capable to sign off a pessary clinician.

The levels have been modified from Benner's Stages of Clinical Competence [11] and amalgamated with the RCOG Core Curriculum Sign off requirements [12] and are described as follows:

- Level 1: Novice (observed practice)—describes a clinician who is at the stage of observation. They observe another healthcare professional carry out a task related to pessary care.
- Level 2: Advanced Beginner (supervised practice)—describes a clinician who performs adequate pessary care under supervision.
- Level 3: Competent (independent practice)—describes a clinician who independently achieves each learning objective, whose practice is clearly underpinned by a sound understanding and knowledge base of pessary care.

We acknowledge that some clinicians have limited means for assessment so may demonstrate their clinical aptitude by other methodologies such as attendance at a course, completion of an e‐learning module or a reflective account of a particular case. We suggest that if a standard is being achieved in this way there should be a minimum of three different types of evidence to support each standard.

*Standards*

1. Knowledge of the indications and management involved in pessary care
2. Knowledge of how to manage complications of pessaries
3. Knowledge of alternatives to pessaries
4. Removal and insertion of pessaries for routine changes
5. Prolapse assessment
6. Assessment for fitting the first pessary
7. Pessary self‐management
8. Reflective practice

*General expectations*

The clinician should be able to adhere to local and national policies on chaperoning, safeguarding, consent, and infection control procedures. This should be demonstrated throughout each standard.
*Standard 1: Knowledge of the indications for pessary use and management involved in pessary care*

*Rationale*
The pessary clinician will need to understand when a pessary can be offered for prolapse management, understand the different variety of pessaries and the level of care needed.
*Knowledge and understanding*
A competent pessary clinician should be able to demonstrate:

- An awareness of local and national policies for prolapse management
- A good knowledge of types of pessary available in their locality
- An understanding of the indication for onward referral when a pessary is no longer managing the prolapse symptoms or if a particular type of suitable pessary is available elsewhere

*Learning outcomes*
The competent pessary clinician will be able to:

- Introduce a pessary to a woman and explain the benefits and risks
- Describe different types of pessary on offer and the rationale for using the selected pessary
- Offer a woman the option of self‐management of her pessary
- Offer a woman pessary management in the short‐term, such as an interim measure whilst considering/waiting for surgery or during pregnancy
- Reassure a woman that pessaries may be used successfully to manage prolapse in the long term
- Describe to a woman the aftercare and follow‐up that is required for the pessary used
- Educate a woman on when to seek medical advice or help

*Standard 2: Knowledge on how to manage complications of pessaries*

*Rationale*
The pessary clinician will need to understand how to manage a range of pessary related complications.
*Knowledge and understanding*
A competent pessary clinician should be able to demonstrate an understanding of:

- The possible complications related to fitting and trial of vaginal pessary device
- The process for reporting complications of pessary use
- How to deal with a range of complications
- Their clinical limitations of expertise and knowledge, and understand when an onward referral is needed

*Learning outcomes*
The competent pessary clinician will be:

- Able to minimize the risk associated with fitting and trial of vaginal pessary device for pelvic organ prolapse
- Competent to perform a speculum examination
- Competent to undertake vaginal swabs
- Able to manage/advise about the use of vaginal oestrogen
- Competent in recognizing complications and be able to set out a management plan for:‐

− abnormal vaginal discharge
− vaginal infection
− abnormal vaginal and vulval health for example, atrophy/vaginitis/lichen sclerosus
− vaginal or vulval abrasion/ulceration
− unexplained vaginal bleeding
− pain/discomfort
− urinary symptoms including voiding difficulty, retention, incontinence
− bowel symptoms including difficulty opening bowels, constipation or incontinence
− difficult removal of pessary

*Standard 3: Knowledge of alternatives to pessaries*

*Rationale*
The pessary clinician should be able to discuss alternative management options for prolapse, if a pessary fails or if a woman does not wish to have a pessary.
*Knowledge and understanding*
A competent pessary clinician should be able to demonstrate:

- An awareness of local and national policies on the alternatives to prolapse management available to a woman
- An awareness of the referral pathways so that a woman may explore her options and discuss alternative management
- An awareness of the risks of surgical management and indications to delay surgery, such as when a woman feels her family is not complete and would like to have further pregnancies

*Learning outcomes*
The competent pessary clinician will be able to:

- Explore what is important to a woman with regards to her treatment goals
- Discuss the option of “doing nothing” and the risks where relevant
- Offer a woman follow‐up when she has chosen to do nothing initially, and allow her to express any change in the management option chosen
- Explain to a woman that there are different types of surgery which may be offered to manage a prolapse, and this is dependent on the type of prolapse
- Discuss alternatives to a pessary, including pelvic floor muscle exercises or surgery if this falls within the clinician's scope of practice. Consider an onwards referral for further in‐depth discussions (such as failure or success rates of treatments)

*Standard 4: Removal and insertion of pessaries for routine changes*

*Rationale*
The pessary clinician will need to be able to remove and insert a pessary. This will include an examination of the vagina and cervix using a speculum to assess tissue quality before pessary insertion. The pessary clinician needs to be competent in each type of pessary used in their individual clinical practice. It is the responsibility of the pessary clinician to dictate their own learning needs. This training document suggests that pessary removal and insertion at level 3 should be demonstrated at least three times. Demonstrating this skill three times is guidance only. Some clinicians may need more practice to be competent. It is unlikely the clinician will be competent in fewer than three demonstrations.
*Knowledge and understanding*
A competent pessary clinician should be able to demonstrate that they:

- Understand the rationale for removing a pessary
- Understand techniques available in order to remove pessaries
- Know how to insert a pessary and ensure a correct fit

*Learning outcomes*
The competent pessary clinician will be able to:

- Communicate effectively throughout the procedure including demonstration of a pessary to a woman, explaining the benefits and risks, and allowing the patient to handle the appropriate pessary
- Prepare the environment
- Remove the current pessary
- Examine the vagina and cervix (if present) by using a speculum and check the health of the tissue
- Assess for a new size if the current pessary does not provide a comfortable fit or if the prolapse is not supported
- Understand when a pessary can be reused
- Insert a pessary
- Test for correct fit of the pessary
- Ensure clear documentation of size and type of pessary that has been fitted
- Know how to refer onwards if clinically indicated

*Standard 5: Prolapse assessment*

*Rationale*
A clinician who is competent in all aspects of pessary care must first be able to accurately assess the presence, compartment involved and degree of pelvic organ prolapse. They should be able to communicate their clinical findings using a standardized and recognized method of reporting, such as the POP‐Q system or Baden‐Walker system. This clinical information will help to guide the clinician and/or healthcare team into choosing the most suitable form of pessary for the woman.
*Knowledge and understanding*
A competent pessary clinician should be able to demonstrate:

- Sound knowledge of female pelvic organ anatomy, including general anatomy of the pelvic floor
- Knowledge of an appropriate recognized grading system for prolapse; being able to competently document their own clinical assessment and also interpret another clinician's findings
- Knowledge of techniques to exclude any pelvic or abdominal masses, which include abdominal and bimanual examination, or refer on if this is beyond practitioner scope
- Techniques used to assess the presence, compartment and degree of prolapse using visual assessment and internal examination
- Awareness of their own limitations and the necessity of clinical support to ensure a complete assessment
- Proficiency in explaining their clinical findings to the individual woman and relate the results to her symptom profile, providing a clear plan for future management

*Learning outcomes*
The competent pessary clinician will be able to:

- Examine for presence of prolapse, assessing which compartments are involved and to what degree
- Record the clinical findings using a recognized standard method of assessment such as POP‐Q system or the Baden‐Walker system
- Explain the clinical findings to the woman
- Relate the clinical findings to the symptoms (and if this is not possible, to consider alternative investigations or onward referral)
- Use the clinical findings to plan ongoing care or referral
- Seek clinical support where necessary

*Standard 6: Assessment for fitting the first pessary*

*Rationale*
A clinician who is competent in pessary care will build on their initial assessment of a woman's prolapse, assess whether the woman is suitable for management with a pessary and then measure the vaginal dimensions to select a suitable type and size of pessary for the individual woman. Knowledge and understanding A competent pessary clinician should be able to demonstrate a knowledge of:

- Indications, contraindications, and precautions when assessing and fitting pessaries for pelvic organ prolapse
- Signs, symptoms, and management of problems occurring during initial pessary fitting for pelvic organ prolapse
- Local policies and procedures in assessing and fitting pessaries for pelvic organ prolapse, specific to the role of the clinician

*Learning outcomes*

- Explain clearly to the woman the process of assessment for the first pessary and how it differs from an examination for prolapse
- Explain that there will be an initial trial period for the pessary, and more than one fitting may be necessary to find the most suitable pessary
- Examine the vagina and cervix using a speculum
- Assess general vaginal and vulval health including signs of vaginal atrophy and organize treatment accordingly
- Perform an assessment of vaginal dimensions and select a pessary type and size to suit the clinical findings
- Insert the pessary
- Test for successful fit of the pessary and assess general comfort once in situ
- Allow time for the woman to ambulate and pass urine after fitting
- Re‐evaluate and reassess if the first pessary is not suitable or not retained
- Discuss pessary management advice for example, sexual intercourse, support perineum when defecating, managing pessary dislodgement
- Ensure clear documentation of size and type of pessary that has been fitted
- Formulate a management plan for ongoing care and plan for safe change of the pessary in an appropriate environment for example, self‐management (if suitable), GP, or specialist clinic
- Provide information to woman regarding pessary and when to contact if there are any problems
- Seek clinical support where necessary

*Standard 7: Pessary self‐management*

*Rationale*
The pessary clinician will need to be able to offer pessary self‐management when indicated.
*Knowledge and understanding*
A competent pessary clinician should be able to demonstrate:

- The skills to teach pessary self‐management
- The knowledge of the types of pessary suitable for pessary self‐management
- A clear pessary self‐management service structure for routine review (including issuing a patient information leaflet and telephone support), and contingency plan for complications including those out‐of‐hours complications

*Learning outcomes*
The competent pessary clinician will be able to:

- Demonstrate indications and contraindications for pessary self‐management
- Communicate effectively the benefits of pessary self‐management to a woman and how self‐management can be used to suit the woman's lifestyle
- Discuss the relevant anatomy, purpose of the pessary and location of the pessary once in situ
- Teach a woman how to insert and remove a pessary, including being able to demonstrate folding or handling the pessary and discussing different positions
- Discuss pessary care (removal frequency, cleaning procedures, lubrication, and storage)
- Advise on sexual intercourse
- Communicate effectively to a woman warning signs and when to contact her pessary clinician
- Supply written patient information to a woman to supplement pessary self‐management care including telephone support

*Standard 8: Reflective practice*
Reflective practice is the ability to reflect on one's actions to engage in a process of continuous learning. [13] It is an important source of personal professional development and improvement and is helpful in bringing together theory and practice. By using this concept in the training and development of vaginal pessary skills, it allows the learner to take a conscious look at their own clinical practice and experiences (both good and bad), how it affected them and add it to their existing knowledge base and thus reach a higher level of understanding. This can then lead to making positive changes or improvements that can be put into action in their everyday practice. Suggested prompts to structure reflection: [14]

- What key things did you take away or learn from this experience/feedback?
- Was it a positive or negative experience?
- What were the consequences of your actions on the patient, others, and yourself?
- What would you do differently, if anything, next time around?
- How has it impacted on your practice?
- Are there any changes you can apply to your practice?
- How can you apply this to meet any gaps in your knowledge, skills, and understanding?

It is suggested the clinician completes a reflective account to document their learning process. Three reflective accounts have been suggested but the clinician should not be limited to this. The following pictogram may help to guide you during your reflective practice. Example:

1. What key things did you take away or learn from this experience/feedback?
  I learnt that I needed to prepare my clinic space with more different sized pessaries. The appointment took longer as I needed to find out if we had the sizes of pessary that I needed.
2. Was it a positive or negative experience?
  Negative for my remaining patient on my clinic list, but I was able to manage this.
3. What were the consequences of your actions on the patient, others, and yourself?
  I ran late with my next appointment. I felt rushed and I am worried I did not explain everything to the patient before she left. However, I know my patient leaflet covers all of the points I would discuss, and I have asked the patient to contact me if she has any questions.
4. What would you do differently, if anything, next time around?
  Prepare my clinic space with more pessaries
5. How has it impacted on your practice?
  I will have more pessaries with me in clinic, rather than in a stock cupboard in the adjacent clinic.
6. Are there any changes you can apply to your practice?
  Prepare more.
7. How can you apply this to meet any gaps in your knowledge, skills and understanding?
  I now understand it can take a few attempts at getting the sizing correct. I would like to spend more time with my colleagues to find out how others size for a pessary.

*Additional resources*

POGP Advanced Pessary Practitioners Course (POGP course content | POGP).

*Standards logbook*

The following pages are designed to provide you with a personal logbook to evidence your learning and competency as a pessary clinician. Please use this as a tool to enhance your learning, engaging with your supervisors to complete each level of the standards required. It is expected a clinician to take between 3 and 6 months to complete if the clinician is working in a full‐time position with access to pessary clinics. This time will increase for those who do not have access to pessary clinics and may need to organize several sessions to complete the logbook.

*Supervision and assessment*

A supervisor is a healthcare practitioner who performs pessary care regularly as part of their normal job role requirements. This may be a practitioner who runs a pessary clinic, or a Consultant Gynaecologist or Registrar with a special interest in urogynaecology or prolapse management. This supervisor will be assessing the pessary care given by the trainee practitioner.

For Level 1—the supervisor is usually the person who is carrying out the pessary care, with the trainee practitioner observing how to complete the task.

For Levels 2 and 3—the trainee practitioner is the person who is carrying out the pessary care with the supervisor observing their practice. For Level 3, the supervisor is acting as an assessor only and should not have to provide any further input for that particular element of care. We recommend that throughout this logbook, the sign‐off sections (Level 1, 2, and 3) are completed by a minimum of two supervisors to ensure each standard is assessed by more than one qualified practitioner.

*Supervisor details*

Supervisors who are signing this document should complete their details in the table below:

**Table:**

Name	Position	Signature	Date

**Table:**

Standard 1: Knowledge of the indication and management involved in pessary care	Level of competence achieved					
	Level 1		Level 2		Level 3	
	Signature	Date	Signature	Date	Signature	Date
**Learning outcomes**						
1. Introduce a pessary to a woman and explain the benefits and risks						
2. Describe different types of pessary on offer and rationale for using selected pessary						
3. Offer a woman the option of self‐management of her pessary						
4. Offer a woman pessary management in the short‐term, such as when considering/waiting for surgery or during pregnancy						
5. Reassure a woman that pessaries may be used successfully to manage prolapse in the long term						
6. Describe to a woman the aftercare and follow‐up that is required for the pessary used						
7. Educate a woman on when to seek medical advice or help						
**Standard achieved with other methodologies (please list below)**						
			Signature			Date

**Table:**

Standard 2: Knowledge on how to manage complications of pessaries	Level of competence achieved					
	Level 1		Level 2		Level 3	
	Signature	Date	Signature	Date	Signature	Date
**Learning outcomes**						
1. Minimize risk associated with fitting and trial of vaginal pessary device for pelvic organ prolapse						
2. Be competent to perform to examine the vagina and cervix using a speculum and check for the health of the tissues examination						
3. Be competent to undertake vaginal swabs						
4. Be able to manage/advise about the use of vaginal oestrogen						
5. Be competent in recognizing complications and be able to set out a management plan for: abnormal vaginal discharge vaginal Infection abnormal vaginal and vulval health for example, atrophy/vaginitis/lichen sclerosus vaginal or vulval abrasion/ulceration unexplained vaginal bleeding pain/discomfort urinary symptoms including voiding difficulty, retention, incontinence bowel symptoms including difficulty opening bowels, constipation, or incontinence difficult removal of pessary						
**Standard achieved with other methodologies (please list below)**						
			Signature			Date

**Table:**

Standard 3: Knowledge on alternatives to pessaries	Level of competence achieved					
	Level 1		Level 2		Level 3	
	Signature	Date	Signature	Date	Signature	Date
**Learning outcomes**						
1. Explore what is important to a woman with regards to her treatment goals						
2. Discuss the option of “doing nothing” and the risks where relevant						
3. To offer a woman follow‐up when she chooses to do nothing initially, and allow her to express any change in the management option chosen						
4. Discuss the option of pelvic floor muscle exercises and refer on if indicated						
5. Discuss the option of surgery to manage prolapse						
6. Explain to a woman there are different types of surgery which may be offered to manage a prolapse, and this is dependent on the type of prolapse						
7. Explain to a woman that surgery carries risks, a failure rate and a risk of recurrence						
**Standard achieved with other methodologies (please list below)**						
			Signature		Date

**Table:**

Standard 4: Removal and insertion of pessaries for routine changes	Level of competence achieved					
	Level 1		Level 2		Level 3	
	Signature	Date	Signature	Date	Signature	Date
**Learning outcomes**						
1. Communicate effectively throughout the procedure including demonstration of a pessary to a woman, explaining the benefits and risks and allow the patient to handle the appropriate pessary						
2. Prepare the environment						
3. Remove the current pessary						
4. Examine the vagina and cervix (if present) using a speculum and check for the health of the tissues						
5. Assess for a new size if the current pessary does not provide a comfortable fit or if the prolapse is not supported						
6. Understand when a pessary can be reused						
7. Insert a pessary						
8. Test for correct fit of the pessary						
9. Ensure clear documentation of size and type of pessary that has been fitted						
10. Know how to refer onwards if clinically indicated						
**Standard achieved with other methodologies (please list below)**						
			Signature		Date

**Table:**

Standard 5: Prolapse assessment	Level of competence achieved					
	Level 1		Level 2		Level 3	
	Signature	Date	Signature	Date	Signature	Date
**Learning outcomes**						
1. Examine for presence of prolapse, compartments involved, and stage of prolapse						
2. Record the clinical findings using POP‐Q system or Baden‐Walker system						
3. Explain the clinical findings to the woman						
4. Relate the clinical findings to the symptoms (and if this is not possible, to consider alternative investigations or onward referral)						
5. Use the clinical findings to plan ongoing care or referral						
6. Seek clinical support where necessary						
**Standard achieved with other methodologies (please list below)**						
			Signature		Date

**Table:**

Standard 6: Assessment for fitting the first pessary	Level of competence achieved					
	Level 1		Level 2		Level 3	
	Signature	Date	Signature	Date	Signature	Date
**Learning outcomes**						
1. Explain clearly to the woman the process of assessment for the first pessary and how it differs from an examination for prolapse						
2. Explain that there will be an initial trial period for the pessary, and more than one fitting may be necessary to find the most suitable pessary						
3. Examine the vagina and cervix using a speculum, check for the health of the vaginal and vulval tissues, assess for vaginal atrophy and organize treatment accordingly						
4. Perform an assessment of vaginal dimensions and select a pessary type and size to suit the clinical findings						
5. Insert the pessary						
6. Test for successful fit of the pessary and general comfort once in situ						
7. Allow time to ambulate and pass urine after fitting						
8. Re‐evaluate and reassess if the first pessary is not suitable or not retained						
9. Discuss pessary management advice for example, sexual intercourse, support perineum when defecating, managing pessary dislodgement						
10. Ensure clear documentation of size and type of pessary that has been fitted						
11. Formulate a management plan for ongoing care and plan for safe change of the pessary in an appropriate environment for example, self‐management (if suitable), GP, or specialist clinic						
12. Provide information to woman regarding pessary and when to contact if there are any problems						
13. Seek clinical support where necessary						
**Standard achieved with other methodologies (please list below)**						
			Signature		Date

**Table:**

Standard 7: Pessary self‐management	Level of competence achieved					
	Level 1		Level 2		Level 3	
	Signature	Date	Signature	Date	Signature	Date
**Learning outcomes**						
1. Demonstrate indications and contraindications of pessary self‐management						
2. Communicate effectively the benefits of pessary self‐management to a woman and how self‐management can be used to suit the woman's lifestyle						
3. Discuss the relevant anatomy, purpose of the pessary and location of the pessary once in situ						
4. Teach a woman how to insert and remove a pessary, including being able to demonstrate folding and handling the pessary and discussing different positions						
5. Discuss pessary care (removal frequency, cleaning procedures, lubrication, and storage)						
6. Advise on sexual intercourse						
7. Communicate effectively to a woman warning signs and when to contact her pessary clinician						
8. Supply written patient information to a woman to supplement pessary self‐management care						
**Standard achieved with other methodologies (please list below)**						
			Signature		Date

**Table:**

Standard 8: Reflective Practice						
**Record number and dates of each reflective practice here and seek supervisor sign‐off accordingly**						
			Signature		Date

**Table:**

Self‐assessment table Practitioners are invited to keep this self‐assessment table updated for when they feel they have achieved each level of competence	Level of competence achieved					
	Level 1		Level 2		Level 3	
	Signature	Date	Signature	Date	Signature	Date
**Learning outcomes**						
Standard 1: Knowledge of the indications and management involved in pessary care						
Standard 2: Knowledge of how to manage complications of pessaries						
Standard 3: Knowledge of alternatives to pessaries						
Standard 4: Removal and insertion of pessaries for routine changes						
Standard 5: Prolapse assessment						
Standard 6: Assessment for fitting the first pessary						
Standard 7: Pessary self‐management						
Standard 8: Reflective Practice

**Table:**

*Action plans for ongoing progress*	
Supervisors and practitioners may use this space to highlight areas which can be worked on in order to progress through the levels Each entry should have the date, the current level, the suggested action plan and a further date to review whether this has been achieved	
Standard 1: Knowledge of the indications and management involved in pessary care	
Standard 2: Knowledge of how to manage complications of pessaries	
Standard 3: Knowledge of alternatives to pessaries	
Standard 4: Removal and insertion of pessaries for routine changes	
Standard 5: Prolapse assessment	
Standard 6: Assessment for fitting the first pessary	
Standard 7: Pessary self‐management	
Standard 8: Reflective practice	

Additional resources: (Figure B1)

*NICE*

- Pathway for the management of pelvic organ prolapse
  https://pathways.nice.org.uk/pathways/urinary-incontinence-and-pelvic-organ-prolapse-in-women
- Assessing pelvic organ prolapse
  https://www.nice.org.uk/guidance/ng123/chapter/Recommendations#assessing-pelvic-organ-prolapse

Pelvic organ prolapse quantification (POP‐Q) system. [15, 16]

## Appendix C

**Pessary types**

Pessary selection and sizing is often more of an art than a science. However, there are several factors that may be considered when selecting a pessary. These include the shape of the vagina, the severity of the pelvic organ prolapse, whether the patient would like to self‐manage her pessary and if she wishes to commence or maintain sexual activity.

This section will describe each of the pessaries commonly used in the United Kingdom, discussing fitting and removal techniques along with information on materials and sizes for each type. Many pessaries are available in a variety of materials, some which are flexible and others that are rigid. Although, it is considered that some women may need a more rigid pessary to support their prolapse, many consider that a flexible pessary is more comfortable, easier to remove and insert and therefore easier to self‐manage. A silicone pessary being softer may be less likely to erode the vaginal walls.

Women can be sexually active with some types of pessary in situ or they may choose to remove the pessary prior to intercourse. This may be more challenging with certain types of pessary for example the Gellhorn, shelf and Donut pessary.

It is noted that there are regional/local variations in pessary preferences and these may be due to experience, knowledge, availability, and cost. Not all pessary clinicians will use all types of pessary in their routine practice, but an understanding of what is available and why it may be used is essential in managing cases where more conventional pessary types have been unsuccessful (Figure C1).

*Ring pessaries*

*Overview*

Rings are the most used pessaries. They can be fitted for any type of pelvic organ prolapse but may be less successful if the perineum is unable to provide enough support such that the ring is poorly retained or if the upper vagina is narrowed by previous surgery for example, hysterectomy. Sexual activity is possible with the pessary in place as the vaginal space is not filled by the device although either partner may find sexual intercourse uncomfortable and may wish to remove it before sex. Self‐management can commonly involve a ring pessary.

*Materials and sizes*

PVC (vinyl) ring pessaries are latex free devices. The outer diameter can vary from 50 to 110 mm and are available in two materials; the flexible vinyl (PVC) pessary has a wall thickness of 12.5 mm and the rigid polythene pessary has a wall thickness of 7.5 mm. It is recommended by the group that the thicker (12.5 mm) ring pessary should be used in preference to the narrower more rigid (7.5 mm) pessary. These can be replaced as per the local policy or as indicated by the pessary condition.

Silicone rings are also available; some of these contain a steel spring filling to help them keep their shape. These pessaries are more pliable than the PVC pessaries and as they can fold, are often easier to insert and remove so are useful for a woman who is self‐managing. They are available in a variety of sizes from 44 to 127 mm outer diameter. Silicone pessaries are more expensive to buy but can be washed and re‐inserted on many occasions. Different manufacturers recommended a variety of timescales but they range from 20 washes/reviews up to 10 years if the pessary is intact and not visibly damaged. Please refer to the individual manufacturer's recommendations for specific advice. Silicone pessaries are commonly powdered with a food‐grade powder that must be washed off with water prior to insertion. Pessaries that contain a metal spring/core may need to be removed prior to certain investigations for example, MRI scans.

*Fitting*

In order to fit a PVC vinyl ring pessary, it should be compressed to reduce its width or twisted into a figure of eight. It may be helpful to run it under warm water to make it more pliable. The compression can be maintained by making a “tube” with the other hand for it to be gently pushed through during insertion as shown in the illustrations below. The compressed ring is introduced into the vagina and once more than half of the compressed ring has been inserted, it can gradually be released as it is further inserted and will usually end up in the correct position without the need for much further adjustment. It may be pushed upwards with the index finger to locate the front edge behind the symphysis pubis. If a woman has a cervix, it should be ensured that the back edge of the pessary lies behind, and not in front, of the cervix. The correct position is with the posterior edge in the posterior vaginal fornix and the anterior edge behind the symphysis pubis. If the anterior edge sits directly under the symphysis pubis, the ring may be too large and may be uncomfortable or not retained. Once fitted a woman can be asked to cough and stand up to ensure the pessary remains in the correct position.

Ring pessary insertion

Ring pessary removal

*Removal*

To remove a ring pessary, an index finger should be hooked around the anterior leading edge of the pessary to bring it down to the introitus. Once it reaches the introitus, it should be compressed as much as possible and then be very gently eased out of the vagina.

*Frequency of change/check*—see Pessary Review Checklist

*Variations of ring pessaries*

*Silicone folding ring pessary*—these are more pliable than vinyl pessaries and fold in the middle at the notches so are easier to insert and remove, especially in a woman with reduced manual dexterity. Insertion and removal techniques are similar to the vinyl ring but the pessary is folded rather than compressed or twisted. The pessary is inserted with the notches aligned to the front and back, then rotated once in place to position the notches to the sides to avoid the pessary folding and being expelled. It should be rotated back again before removing so that it folds correctly to aid removal.

Folding ring pessary

Ring with knob—is designed for a woman with both pelvic organ prolapse and stress urinary incontinence (SUI) and can also be useful when new symptoms of stress incontinence develop when her prolapse is reduced with an initial trial of a pessary without knob. The knob adds additional width to the pessary which may affect the sizing and provides support to the bladder neck to reduce SUI. It should be inserted like a standard ring but with the knob to one side and then rotated in the vagina so that the knob sits behind the symphysis pubis. It should be rotated back again before removal. For a woman without prolapse but just SUI, an incontinence pessary is available, which is the same design but with a thinner ring.

Ring pessary with knob

Silicone ring with support—for a woman who has a more significant degree of uterine prolapse, for example when the pessary is in place and she is aware of the prolapse protruding through the ring or feels that the pessary is not providing enough support, a ring with support may be considered. It provides a flexible supporting membrane with drainage ports that prevents the uterus falling though the center of the ring.

Ring pessary with support

*Gellhorn pessary*

*Overview*

Gellhorn pessaries are a circular, flat plate with a stem in the center which stabilizes the pessary in the vagina. It is often considered for a woman who has more advanced prolapse or who needs additional support. The cervix or vaginal vault rests behind the flat plate of the pessary and the stem should only be visible at the introitus when the woman performs a Valsalva manoeuvre (strains downwards). Although many women feel that sexual activity is not possible with a Gellhorn in, some women can maintain certain sexual activities. Self‐management is a lot more difficult with this pessary but it is possible.

*Materials and sizes*

Most Gellhorn pessaries are made of a flexible silicone material, however rigid silicone and acrylic varieties are available. They are sized in two ways; the first in the same way as a ring pessary to determine the outer diameter size of the circular plate (available in 38–95 mm or equivalent inches) and secondly the vaginal length must be considered to enable a choice between a standard‐length stem or short stem. The pessary has drainage ports to allow the passage of fluids, although they do not readily allow drainage of menstrual flow.

*Fitting*

There are two options for Gellhorn pessary fitting depending on the estimated size of the Gellhorn and the size of the introitus:

1. Holding it with the stem flattened sideways to compress the pessary, the edge of the plate is introduced first; once half of the plate is inside the vagina, the pessary is then rotated into a horizontal position whilst pushing it upwards at the same time so that the edge is placed in the posterior fornix with the stem sitting in the center of the vagina.
2. Folding the plate of the pessary behind the stem, the pessary is then introduced into the vagina and pushed towards the posterior fornix, with the stem sitting in the center of the vagina.

Gellhorn pessary insertion technique 1—stem folded

Gellhorn pessary insertion 2 ‐ plate folded

Gellhorn pessary in situ

Gellhorn pessary removal

*Removal*

A finger needs to be introduced to the side of the plate to move it and release the suction. Once the plate is mobile, the pessary should be folded by placing the middle finger around the stem. The index finger is then hooked round the edge of the plate folding it towards the stem whilst also bringing it down to the introitus. The tip of the stem needs to be outside the introitus before the plate can be gently eased out compressing the edges if possible, to reduce the diameter.

A Gellhorn pessary can be difficult to remove but the following may be tried:

- using a sponge holder on the stem to allow better grip and then easier access to slide your finger around the back to release the suction.
- a sponge holder can also be applied to the edge of the pessary to help bring it down.
- use of a speculum if the pessary is sitting high in the vagina can be helpful to find the stem to put the sponge holder onto to help remove it.
- water or a local anaesthetic gel can also be inserted through the stem of the pessary to help break the suction of the plate and facilitate removal without discomfort.
- asking the woman to lift her bottom slightly (bridging) or to roll onto her side can help release the suction and increase the space for easier removal

*Frequency of change/check—see pessary review checklist*

*Shelf pessary* (Figure C3)

*Overview*

Shelf pessaries have a kidney‐shaped plate for support with a curved stem in the center for stabilization in the vagina. The convex edge of the pessary sits in the posterior vaginal fornix and the concave edge faces toward the bladder. It is usually used for a woman with more advanced prolapse. Challenges with sexual activities and self‐management are the same as for the Gellhorn pessary.

*Materials and sizes*

A shelf pessary may be rigid and not compressible (made of an acetyl copolymer) or made of silicone and compressible which makes removal easier. They are available in a range of different sizes (51–102 mm for the rigid and 38–95 mm for silicone). The silicone pessaries are also available in a standard and short stem length. The pessary has drainage ports to allow passage of fluids, although they do not readily allow drainage of menstrual flow.

*Fitting*

It is fitted by holding it firmly with the stem pointing sideways so that the thin edge of the plate is introduced first. Once half of the plate is inside the vagina, the pessary is then rotated into a horizontal position whilst pushing it upwards at the same time so that the posterior round edge is placed in the posterior fornix. The stem should point forward.

Shelf pessary insertion

Shelf pessary removal

*Removal*

The index finger is hooked behind the edge of the plate to release the suction that builds up between the pessary and the vaginal walls allowing the pessary to be brought down so that the tip of the stem is outside the vagina. Once the stem is outside, the pessary may be rotated to place the plate in a vertical position and ease it out of the introitus. Either the anterior or the posterior edge may be released first depending on which is easier.

*Frequency of change/check—*see pessary review checklist

*Shaatz pessary*

*Overview*

The Shaatz pessary is similar to the Gellhorn pessary but without a stem. It is recommended for a woman with a low or shallow pubic notch who cannot retain a ring pessary. It is ideal for a woman who wishes to maintain sexual activity but requires more support. It is suitable for self‐management.

*Materials and sizes*

Shaatz pessaries are generally made of soft silicone and have drainage ports to allow the passage of fluids. Shaatz pessaries are also called folding Shaatz as they can fold in half making insertion and removal easier. They are available in a range of sizes between 38 and 95 mm outer diameters.

*Fitting*

The Shaatz pessary is fitted in a similar way to a ring pessary. The concave side is towards the top of the vagina to allow mild suction.

Shaatz pessary insertion

Shaatz pessary removal

*Removal*

The pessary is removed by inserting one finger to the side of the plate to move it and release the suction and then into the large hole to bring the pessary down toward the introitus. The pessary is then turned so that the rim is almost parallel to the introitus. With one or two fingers of the other hand, press down on the perineum and slide the pessary out. For removals that are difficult, try tying a long piece of dental floss through the ports of the pessary and use this to pull down to allow for an easier removal.

*Frequency of change/check—*see pessary review checklist

*Cube/tandem cube* (Figure C5)

*Overview*

The cube pessary has six concave sides that create a suction effect when in place in the vagina helping it to be retained. It is therefore often used in cases of more severe prolapse where other pessaries have failed. It is only suitable for a woman who can self‐manage as it needs to be removed and cleaned daily. The woman will need a degree of manual dexterity to be able to manage insertion and removal. The pessary will need to be removed prior to sexual activity involving vaginal penetration.

*Materials and sizes*

The cube is made of silicone and available in a variety of different sizes ranging from 25 to 75 mm and is also available with or without drainage holes. The pessary would be washed with mild soap and water each night. Some cubes have drainage ports to allow the passage of fluids. Although they do not readily allow passage of menstrual flow.

*Fitting*

To insert it into the vagina, the cube should be pushed gently downwards into the vaginal space and then with downward pressure to the posterior vaginal wall whilst being turned gently to get past the vaginal entrance. Once inside the vagina, it should be pushed up as far as possible.

Cube pessary insertion

Cube pessary removal

*Removal*

Daily removal is considered advisable.

The string of the pessary is used to help locate the base of the pessary. The string should not be used to pull the cube down and out.

A finger should be inserted into the vagina to sweep around the pessary and move it gently to break the suction. Gentle bearing down if required may help to get a firmer grasp of the pessary. Twisting the cube slightly to ease it out of the introitus may be helpful.

*Frequency of change/check*

These need to be removed daily. Assessments by a HCP are routinely performed after the first 4–8 weeks of use and then at 3–6 monthly intervals thereafter to check the health of the vaginal walls.

*Variations of cube pessaries*

*Tandem cube*

This provides additional support for a woman who is unable to keep the largest size cube pessary in place. It has 10 concave sites increasing the overall suction of the pessary to increase adhesion to the vaginal walls.

*Inflatable pessaries*

*Overview*

Like the Donut pessary, the inflatable pessary works by occupying the space in the vagina. The pessary consists of the head, which is inserted into the vagina, and the stem which sits outside of the body. The stem has a bead in the closed end which controls the inflation and deflation. A separate bulb (hand pump) is attached to the open end of the stem for inflation. An advantage of the inflatable design is that it is inflated once it is in situ, thus making insertion more comfortable for some women. However, this pessary needs to be removed and cleaned every night so a woman must be willing to self‐manage. The pessary can be removed prior to sexual activity. The inflatable pessary should not be left in place for more than 24 consecutive hours.

*Materials and sizes*

Inflatable pessaries are made of latex or silicone. They are available in four sizes ranging from 51 to 70 mm. The latex inflatable pessaries cannot be used in a woman with a latex allergy and should not be used with any vaginal hormone creams as these contain a wax base that will deteriorate the latex.

*Fitting*

The pessary is deflated by moving the bead to the closed end of the stem. The bulb is then attached into the open end of the stem. The head of the pessary is then inserted into the vagina so that only the stem protrudes. The pessary is inflated by squeezing the bulb and the inflation level is controlled by the number of pumps. Once inflated the bead in the stem is moved forward to close the air vent and the bulb removed. The stem can either be tucked into the vagina or left outside.

Inflatable pessary insertion

Inflatable pessary removal

*Removal*

The bead is moved into the closed end of the stem allowing the pessary to deflate. Using the stem as a guide the woman can grasp the deflated pessary and remove it. The stem should not be used to pull the pessary out.

*Frequency of change/check*

A woman needs to remove and wash the pessary with warm soapy water daily. HCP's will usually review after the first 4–8 weeks of use and every 6–12 months thereafter.

*Vaginal Dish pessaries with knob/urethral bowl*

*Overview*

Dish pessaries are a circular vaginal pessary, with a cupped shallow bowl (with or without support) with a knob that is designed to lift the bladder neck. This elevation can reduce symptoms of stress urinary incontinence but as it can sit nicely behind the pubic bone it can also work effectively in supporting a large anterior prolapse with or without uterine descent where a ring has failed, whether there is incontinence or not. This can be useful, when a woman does not want a more complex pessary such as a Gellhorn, particularly if she wants to remain sexually active. Self‐management is possible with this pessary.

*Materials and sizes*

Pessaries are made of a soft flexible silicone material. They are sized in a similar way to a standard ring pessary but the knob will add to the general diameter (55–90 mm) so a smaller diameter may be needed against normal measurement. There is, however, some manufacturer variation of roundness of the knob and careful consideration needs to be given to some that have a squarer knob that provide less support.

The pessary with support has drainage ports to allow the passage of fluids. Although they do not readily allow passage of menstrual flow.

*Fitting*

The pessary is fitted by holding the two edges of the diameter with the knob at the top to compress the pessary. The opposite edge of the knob is inserted first towards the posterior fornix pushing the knob up behind the pubic bone.

*Removal*

The pessary is removed by inserting one finger into the large hole under the knob to bring the pessary down toward the introitus. Alternatively, put a finger over the top of the knob first and then remove. The pessary is gently then pulled down and the edges will generally collapse as it is removed.

*Frequency of change/check—see pessary review checklist*

*Other pessaries*

There are several other types of pessary available in the United Kingdom that have not been included in this section as they are very rarely used. These include (but are not limited to) the Donut, Gehrung, Hodge, Smith, Risser, Marland, Regula pessaries. Specific information related to these products can be found in the manufacturer's instructions.

*Acknowledgment*

With thanks to Mediplus UK for permission to use the images of pessaries in this appendix

## Appendix D

**Patient information**

*Information for women using a pessary for vaginal prolapse*

*Using a pessary for vaginal prolapse*

This leaflet explains what vaginal prolapse is, the benefits and the risks of having a pessary for treatment of vaginal prolapse, and the alternatives to pessaries.

We have answered the most common questions asked by women regarding prolapse and pessaries. However, we do understand that every woman's situation is different and individual to them and advice from an appropriate healthcare professional is always preferable. If you do have any specific questions, it is important to speak to your own doctor, nurse specialist or physiotherapist for further help.

*What is a vaginal prolapse?*

Vaginal prolapse is a common condition where the walls of the vagina and sometimes the uterus (womb), or vaginal roof (if you have had a hysterectomy) bulge downwards towards the entrance of the vagina. A vaginal prolapse is also known as a pelvic organ prolapse.

*What are the symptoms of a vaginal prolapse?*

You could have one or more of these symptoms:

- A feeling of something coming down, a dragging sensation or a bulge in or out of the vagina
- Difficulty emptying your bladder or bowel
- Urinary urgency
- Discomfort/pain during sexual intercourse
- Discomfort or aching in the pelvis and low backache

*What increases the risk of vaginal prolapse?*

There are a number of factors that might increase a woman's risk of prolapse. Some factors cannot be changed—such as ageing and genetics.

- Childbirth—with increased risk from a vaginal delivery and the use of forceps for delivery
- Getting older
- Being overweight
- Persistent coughing
- Constipation
- Persistent Heavy lifting such as in a manual occupation, or prolonged standing
- Genetic history

*What can be done to help improve your symptoms?*

The following are sometimes referred to as “conservative” or “non‐surgical” approaches.

- Lifestyle changes that may include
  ◦ Losing weight,
  ◦ treating constipation and avoiding straining on the toilet
  ◦ reducing or modifying heavy lifting activities to reduce the strain on your pelvic floor muscles and supporting structures.
  ◦ Exercise and activity—staying active and possibly adjusting the type and timing of exercise to avoid worsening symtpoms

- Using a vaginal pessary to support the prolapse when exercising and keeping PFM active.
- *Vaginal health* including use of topical oestrogen and appropriate vaginal moisturizers and lubricants
- Pelvic floor muscle exercises help to strengthen muscles that are weak, and PFM training may help to improve the support the PFM can give to (Figure C2). your pelvic organs—bladder, bowel, and uterus
  ◦ Information about pelvic floor muscle exercises: https://thepogp.co.uk/patients/pelvic_health_advice/pelvic_floor_muscles.aspx
  ◦ Audio pelvic floor muscle exercise guide: https://www.youtube.com/watch?v=92KUPKi-ii4&feature=emb_logo

If you have difficulty doing the exercises, are worried that you might not be getting them right or find that your symptoms are not reducing, you can ask to be referred to a pelvic health physiotherapist.

*In some cases, surgery may be recommended. Surgery is offered when other nonsurgical options haven't managed to control or reduce your symptoms. This will be a shared decision with all the pros and cons explained.*

The NICE healthcare guideline recommend that you see a pelvic health physiotherapist for a minimum of 4 months before considering surgery for a small or medium sized prolapse. The specialist physiotherapist will help you work out what lifestyle measures may work for you, and teach you how to correctly use your pelvic floor muscles to support the prolapse and reduce the bothersome symptoms. Not all women with a prolapse have weak pelvic floor muscles, and sometimes the physiotherapist will help you to relax the muscles and allow the body to work more naturally.

*What is a vaginal pessary?*

A vaginal pessary is a device, made of plastic or silicone, which is inserted into the vagina to hold a prolapsed uterus or vaginal wall in place. It will also support your bowel and bladder. There are different types of pessaries and the healthcare professional who assesses your prolapse will discuss the type best suited to you. It sometimes takes more than one visit to get the right size, fit and type for you.

*What are the benefits of a vaginal pessary?*

Once fitted correctly a vaginal pessary may help to reduce your symptoms and make you feel more comfortable. You will be able to continue with your everyday activities including exercising, working and caring for your family.

*What are the risks of a vaginal pessary?*

There are a few side effects and risks. Your healthcare professional will tell you about these.

- You may notice you have more vaginal discharge than normal. If the discharge is offensive see your pessary fitting clinician or your GP.
- Occasionally bladder or bowel function could be affected.
- You may have vaginal irritation. If you feel sore and are suffering from symptoms associated with vaginal atrophy or have been through the menopause, you may benefit from using vaginal oestrogen.
- Long‐term use of a vaginal pessary may cause ulcers (sores) inside the vagina, and/or infection. Ensure to follow advice on pessary timescales see Pessary Review Checklist. You may also be prescribed vaginal oestrogen.

*Vaginal oestrogen* can be used either as a cream or in the form of a pellet (also called a pessary) or a slow release ring which is inserted into the vagina. The hormone is absorbed by the vaginal tissue and only works locally. This means the amount absorbed into the blood stream is minimal and therefore should not be confused with Hormone Replacement Therapy (HRT) which is given for more general menopausal symptoms. Your healthcare professional will advise you as to which vaginal oestrogen is most suitable for you.

Vaginal health is an important aspect of pessary success. It can help with pessary comfort, increase the chance of longer‐term pessary use, and make pessary change appointments more comfortable.

If you have been advised to use vaginal oestrogen, it is important that you keep up with regular use, even if you are taking general HRT for menopausal symptoms.

Vaginal moisturizers, which can be bought over the counter, will improve any general vaginal dryness without the need for vaginal oestrogens but will not treat vaginal atrophy. They can be used alongside prescribed vaginal oestrogen or in isolation in the absence of vaginal atrophy.

*Patient information: Self‐Management of Vaginal Pessary*

Infographic: Self‐management of vaginal pessary

*What do I need to know?—information for women about self‐management using a pessary for prolapse*

- How do I remove my ring pessary/How do I remove my cube pessary?
- How do I clean +/‐ store my pessary?
- How do I insert my ring pessary/How do I insert my cube pessary?
- How long can I continue using my pessary?
- What problems should I look out for?
- What do I do if I have any problems?

*To self‐manage your pessary, you need to:*

- Want to self‐manage
- Have the ability and confidence to remove and reinsert the pessary
- Be aware of how to look after the pessary e.g. checking its condition and cleaning the pessary
- Be able to re‐order a new pessary or request one on repeat prescription
- Monitor any changes and recognize when help or advice might be needed

*Removing and reinserting the pessary*

- The person who assessed you and fitted your pessary will have shown you how to put the pessary in and take it out. Help will be available until you feel confident to do this on your own.
- You will be advised how often to remove the pessary depending on the type of pessary you are given.
- For pessaries that don't need to be removed daily there are no hard and fast rules on how long you keep a pessary inserted, but as a minimum it is recommended that you remove it every 3 months.
- There is no harm in removing your pessary and leaving it out for a period of time if you wish to. For example: you could remove it overnight then wash and reinsert in the morning about once a month.
- You could just use it for exercise if that is the only time your prolapse bothers you.
- You can discuss any questions you have about removing your pessary with the person who provided you with the pessary.

*Cleaning the pessary*

You will be advised how to clean your pessary. It usually involves washing with warm water and a mild pure soap. There is no need to sterilize the pessary. Sterilizing can have an adverse effect on the material that the pessary is made from. When cleaning your pessary, it is a good idea to check its condition. Look for any cracks or splits in the material that might have appeared or a general change in the pessary's condition. Discolouration of the pessary often occurs and is not harmful.

*Changing the pessary*

You will be told at the clinic where your pessary was fitted, whether you should either order a new replacement pessary from the clinic directly or request it on repeat prescription. As a guide the minimum time for replacing the pessary with a new one is once every 6 months.

*Tips to help with any changes to your condition*

- If you have difficulty emptying your bladder or bowel remove the pessary first and once you have successfully passed urine or opened your bowels wash the pessary and reinsert it. If this happens regularly contact your clinician as you might benefit from a smaller size pessary.
- Avoid getting constipated
- Supporting the area between the anus and the opening of the vagina (Figure C4) when opening your bowels gives confidence to push without pushing the pessary out. Wrap toilet paper or a sanitary pad around your hand and press and support the area just in front of the anus and around the opening of the vagina.

- Occasionally a pessary may come out completely and you may need to wash and reinsert it or replace with a new one. If this happens often contact your clinician as you might need a bigger size of pessary.
- It is normal for the pessary to move sometimes. This may cause discomfort. If this happens, try moving the pessary back into position.
- It is a good idea to have a spare pessary available.

*If any of the following symptoms occur, you should contact the clinic or your GP*

- *Smelly or offensive discharge*. An increase in discharge is very common but it should not be smelly. If you notice smelly discharge, contact your clinic or GP. You can change pessaries more often to help with discharge.
- *Bleeding from the vagina*. If this happens, take the pessary out if you are able to and contact your clinic, healthcare professional, or GP.
- *Soreness, discomfort, or pain*. If this happens, take the pessary out if you are able to and contact your clinic or GP.

*Frequently asked questions*

*Who will fit my pessary?*

A qualified healthcare professional with experience in managing pessaries.

*Can I ask for a female practitioner?*

Yes, you can, although there may not be a female practitioner available in every clinic so you may want to make this request in advance of your appointment. You should be offered a chaperone and you may also choose to have someone else with you such as a partner, friend, or relative.

*Will I get a choice of device, or see what my pessary looks like before it is fitted?*

Not all pessaries work for all women and all types of prolapse. After you have been assessed, the person who is fitting your pessary should show you which device is most likely to be appropriate for you. They will discuss your options with you, which may include the option of managing the pessary yourself. They will then show you the device, explain how it works and fit your pessary with your consent.

Please note: Sometimes it can take more than one fitting to find the device and size that works best for you. Throughout the process your doctor, nurse practitioner, or physiotherapist will work with you to make sure that you get the best possible results.

*How do you know what size to fit? What does it look like?*

The person fitting your pessary will have assessed which size pessary is required and will show the pessary to you before it is fitted. Ask to see what it looks like.

*What is it made of?*

Pessaries are usually made of plastic or silicone.

*Will I feel it in place?*

No, ideally you should not feel it once it is in place and have a good fit. However, the pessary can move within the vagina, a bit like a tampon, so you may be aware of it at times, but it should not be uncomfortable. The person fitting your pessary will be able to show you what to do If it does become uncomfortable.

*Will it fall out when I exercise?*

A pessary should not fall out if it fits correctly. There is an element of trial and error when fitting pessaries for the first time, so a pessary could fall out while you are trying different ones. Once you have a well‐fitting and comfortable pessary, it will not come out during exercise, but it can still move.

*Should I give up my exercise activities?*

No. You should be able to continue exercise with a pessary. Many women find they can be more active once they have a correctly fitted device in place.

*I have been doing pelvic floor exercises. Should I continue with them?*

Yes. It is important to continue with your pelvic floor exercises.

*Can I use a tampon?*

Yes. You can use a tampon if you have a ring type pessary as long as it feels comfortable.

*Will it fall out when I strain to open my bowels?*

Supporting the area between the anus and the vagina when opening your bowels gives confidence to push, without pushing the pessary out. Wrap toilet paper or a sanitary pad around your hand and press and support the area just in front of the anus and around the opening of the vagina. You can also push/hold on to the pessary while having a bowel movement. Avoid getting constipated.

*How often should a pessary be checked or changed?*

Depending on which type of pessary you have, this can be every 3–6 months by a healthcare professional. For self‐managing pessaries, how often you remove a pessary depends on the type of pessary you were given and personal choice, but it is still important to check your pessary regularly. For pessaries that don't require removing daily, they can be removed occasionally, for example once a week or a month, though you do not have to do this if you don't want to. As a guide the minimal time for changing a pessary and replacing it with a new one is once every 6 months.

*Will I be prone to more vaginal infections?*

You may occasionally get a vaginal infection which can be treated with a local antibiotic vaginal cream or by removing the pessary and leaving it out for a while. Oral antibiotic tablets will be given if required.

An infection is less likely if the pessary is changed regularly and the vaginal tissues are kept healthy by using prescribed vaginal oestrogen or the use of moisturizers.

*Will it affect my sex life?*

It is possible to have penetrative vaginal sex with some pessaries such as a ring or Shaatz pessary, but others would have to be removed first and replaced later. You will be able to discuss with your healthcare professional options of which pessary might best suit you, along with any concerns you may also have about contraception.

*How difficult is it to remove and put back in?*

Some pessaries can be self‐managed. The clinician who fitted your device will assess suitability and discuss this with you and will teach you how to remove it and reinsert it while you are with them in clinic. It is not usually difficult to remove a pessary or replace one once you have been shown how and have practiced.

*Can I self‐manage my pessary?*

Yes. You may be offered self‐management of your pessary by your healthcare professional if suitable. You can manage your pessary yourself once you have been shown how to, have practiced and are confident to do so.

*Do I need to use lubricants?*

You may find it more comfortable to use a vaginal lubricant when inserting a pessary, especially a new one, but you do not have to.

*What is vaginal oestrogen and why might I need it?*

Vaginal oestrogen can be used either as a cream or in the form of a pellet (also called a pessary) or a slow release ring which is inserted into the vagina. The hormone is absorbed by the vaginal tissue and only works locally. This means the amount absorbed into the blood stream is minimal and therefore should not be confused with Hormone Replacement Therapy (HRT) which is given and absorbed systemically for more general menopausal symptoms. Your healthcare professional will advise you as to which vaginal oestrogen is most suitable for you.

Oestrogen is found naturally in the body and one of its functions is to keep the condition of the vaginal tissues healthy. Oestrogen may be reduced during and after the menopause and when breast feeding and it can cause vaginal atrophy. Vaginal atrophy is when the lining of the vagina becomes thin and dry and the skin around or in the vagina can feel sore or itchy. Your healthcare professional will advise you if you need oestrogen and which type will be most suitable for you.

*How effective will vaginal oestrogen be?*.

Vaginal or topical oestrogen can be very effective at making the condition of the vagina healthier and reducing soreness and discomfort and in turn, reducing the risks of infection and ulcers which a vaginal pessary may cause. It may be beneficial to use vaginal oestrogen on a daily basis for 7–10 days prior to your pessary change appointment.

*Can I expect my prolapse condition to go back to normal after a while or is this it for the rest of my life?*

No. Using a vaginal pessary controls the symptoms of a prolapse by supporting it. The pessary will not ‘heal’ or ‘cure’ the prolapse. Sometimes when the pessary is removed, there may be a temporary improvement, but this is not likely to last over time.

*Is this currently the preferred treatment for prolapse?*

It is one of a small number of choices available for managing vaginal prolapse, but each woman is different and treatment choice varies with each individual. You can change your mind about your treatment at any time.

*My friend had surgery and that was fine for her. This was not offered to me as an option. What are my options?*

Surgery may be considered but often women are offered a less invasive option to try first as all surgery will carry some risk including prolapse recurrence. Surgery for prolapse may include hysterectomy if the uterus has prolapsed. However, with some surgical procedures women do not need to have a hysterectomy. Removing the uterus itself does not always cure the prolapse and may make other types of prolapses more common.

*Can I buy a pessary from my local chemist myself to experiment myself?*

It is possible to buy some pessaries online but it is not advisable without having been assessed by a trained professional who can make sure you have the correct and most suitable pessary. There are a large variety of pessaries on the market and an appropriately trained healthcare professional will be able to help you find one that is likely to help you and your specific prolapse and symptoms. If you buy one online, it would be advisable to ask a healthcare professional to check it is suitable for you before you use it.

*What happens if I am self‐managing my pessary and it gets stuck?*

Don't panic! It is much easier to remove a pessary if you are relaxed and in the right frame of mind.

Contact the clinic where the pessary was fitted, your GP or go to the nearest A&E.

**Table:**

Useful contact details
Your clinic is:
Contact telephone number:
Your pessary is:
Fitted on:
For type of prolapse:

*Useful reading*

- The Pelvic Obstetric and Gynaecological Physiotherapy website https://thepogp.co.uk
- Vaginal Prolapse https://thepogp.co.uk/patient_information/womens_health/vaginal_prolapse.aspx
- Pelvic organ prolapse: a physiotherapy guide for women https://thepogp.co.uk/_userfiles/pages/files/POGP-Prolapse_2.pdf
- Pelvic floor muscle exercises for women https://thepogp.co.uk/_userfiles/pages/files/POGP-PelvicFloor%20(UL).pdf Appendix C—Patient information

*Copyright*

Reproduction of any individual part of each section including illustrations without permission is not allowed.

Copying and further use guidelines (including images) can be found on the POGP/UKCS website.

## Appendix E

**Updated search strategy adapted from 2021 pessary guidance document**

**Table:**

Databases	Medline, Cochrane library databases (Cochrane Database of Systematic Reviews—CDSR, Health Technology Assessment—HTA, Database of Abstracts of Reviews of Effects—DARE, Cochrane Central Register of Controlled Trials—CENTRAL), CINAHL (EBSCO), PsycINFO (EBSCO)
Inclusion criteria	“pessaries” “quality of life” “pessary” “prolapse”, “confidence” “training” “healthcare professional” and “competency” “pelvic organ prolapse”
Exclusion criteria	Nil
Study design	Not limited to any type
Years	2018–2025
Limitations	English language
